# The longstanding issue of the benefit of ursodeoxycholic acid in primary sclerosing cholangitis: a long-term population-based study

**DOI:** 10.1007/s00535-026-02449-7

**Published:** 2026-06-15

**Authors:** Bregje Mol, Kim van Munster, Rinse Weersma, Akin Inderson, Kirsten Boonstra, Joost Drenth, Suzanne van Meer, Elsemieke de Vries, Marcel Spanier, Anton Vrij, Tessa Verlaan, Femke van Duijn, Willemijn Ponsioen, Marcel Dijkgraaf, Cyriel Ponsioen

**Affiliations:** 1 Department of Gastroenterology and Hepatology, Amsterdam University Medical Centre, Amsterdam, The Netherlands; 2 Amsterdam Gastroenterology, Endocrinology & Metabolism, Amsterdam, The Netherlands; 3 Department of Gastroenterology and Hepatology, University of Groningen and University Medical Center Groningen, Groningen, The Netherlands; 4 Department of Gastroenterology and Hepatology, Leiden University Medical Center, Leiden, The Netherlands; 5 Department of Gastroenterology and Hepatology, Radboud University Medical Center, Nijmegen, The Netherlands; 6 Department of Gastroenterology and Hepatology, University Medical Center Utrecht, Utrecht, The Netherlands; 7 Department of Gastroenterology and Hepatology, Isala Clinics, Zwolle, The Netherlands; 8 Department of Gastroenterology and Hepatology, Rijnstate Hospital, Arnhem, The Netherlands; 9 Department of Gastroenterology & Hepatology, Zorggroep Twente, Almelo, The Netherlands; 10 Department of Gastroenterology & Hepatology, Gelderse Vallei Hospital, Ede, The Netherlands; 11 Department of Epidemiology and Data Science, University of Amsterdam, Amsterdam Public Health, Amsterdam University Medical Center, Amsterdam, The Netherlands

**Keywords:** Primary sclerosing cholangitis, Ursodeoxycholic acid, Observational study, Long-term benefit

## Abstract

**Background:**

Although ursodeoxycholic acid (UDCA) is commonly prescribed for primary sclerosing cholangitis (PSC), a beneficial long-term effect on solid clinical endpoints has not yet been established. This study evaluated the efficacy of UDCA on long-term transplant-free survival in a longitudinal population-based cohort.

**Methods:**

We conducted a retro/prospective study between January 2008 and August 2020, using data from the Dutch population-based EpiPSC2 cohort. Medication use was collected through medical chart review, periodic questionnaires, and the national pharmacy prescription database. The effect of UDCA use was assessed as time-dependent variable using a Cox proportional hazards model. Sex, age at diagnosis, PSC type, IBD status, transplant center inclusion, and PSC diagnosis year were considered as covariates.

**Results:**

Medication data were available for 739 cases. Individuals with uncertain use of UDCA were excluded, resulting in a study population of 527. No discernable effect of UDCA use was observed on the endpoints liver transplantation (LT)/all-cause mortality [adjusted HR 1.13 (95%CI 0.76–1.67)] nor LT/PSC-related death/occurrence of hepatobiliary malignancy [adjusted HR 1.07 (95%CI 0.71–1.60)]. The HR of UDCA use was numerically reduced for hepatobiliary malignancy [0.68 (95%CI 0.34–1.37)].

**Conclusion:**

Our study failed to demonstrate that UDCA use impacts long-term outcomes in patients with PSC.

**Supplementary Information:**

The online version contains supplementary material available at https://doi.org/10.1007/s00535-026-02449-7.

## Introduction

Primary sclerosing cholangitis (PSC) is an enigmatic cholestatic liver disease affecting 6–32 individuals per 100,000 [1, 2]. PSC is strongly associated with inflammatory bowel disease (IBD) and patients are at increased risk for developing hepatobiliary (HB) malignancies, as well as colorectal cancer (CRC) when IBD is present [1]. Disease course is highly variable and there is no treatment available with proven efficacy in halting disease progression other than orthotopic liver transplantation (LT). In several European countries, PSC nowadays belongs to the top three indications for LT. In the largest population-based cohort to date the median transplant-free survival time was 21.2 years [1]. This implies that 50% or more of PSC patients is either dead or in need of a liver transplant before the age of 60, a notion which has affixed PSC with the dubious reputation of being killer number one in IBD. None of the immunomodulatory and antifibrotic agents studied in PSC so far have been shown to halt disease progression [3]. The only drug that is used due to its cytoprotectant and choleretic properties is ursodeoxycholic acid (UDCA). UDCA conjugates exert anticholestatic effects and stimulate biliary bile acid and organic anion secretion by mainly post-transcriptional molecular mechanisms at the level of the hepatocytes [4]. Possibly more relevant for PSC, UDCA conjugates also stimulate biliary bicarbonate secretion at the level of cholangiocytes and hepatocytes leading to stabilization of a ‘biliary bicarbonate umbrella’ [5], impaired in PSC [6], and, thereby, prevent carrier-independent invasion of apolar, potentially proliferative hydrophobic bile acids into cholangiocytes [7]. However, 2 decades of clinical research, including post hoc analyses of randomized trials, has failed to provide evidence that UDCA use improves transplant-free survival [8]. Despite the lack of evidence and the absence of formal approval by the regulatory authorities, many patients with PSC are treated with UDCA. The European Association for the Study of the Liver (EASL) guidelines suggest UDCA at 15–20 mg/kg/d noting limited evidence but citing potential improvement in liver tests and surrogate markers of prognosis [9]. The 2019 British Society of Gastroenterology guideline does not recommend UDCA, while the 2022 American Association for Study of the Liver Practice Guidance is also open to treatment with UDCA if there is biochemical response [10, 11].

Our limited knowledge of PSC is in part due to its low prevalence and the infrequent occurrence of solid clinical endpoints. These have an estimated annual event rate of 5% requiring lengthy follow-up that is hardly feasible for clinical trials. Moreover, most cohorts come from tertiary centers, which inherently are prone to referral bias. To counter these limitations we have initiated in 2008 the population-based Epi PSC/PBC project [12]. Together with data from the national pharmacy prescription database (Stichting Farmaceutische Kerngetallen, SFK), this population-based registry provides a unique opportunity to study the long-term effects of commonly prescribed drugs in individuals with PSC with regard to their effect on the natural history. The aim of this current study is to assess the effectiveness of UDCA on transplant-free survival on a patient level in a long-term population-based observational study.

## Patients and methods

### Study design and population

The study was conducted with data arising from the EpiPSC2 study, which is a dynamic registry consisting of individuals with PSC in The Netherlands [1, 12]. The registry started in 2008 as population-based EpiPSC/PBC study that comprised retrospective data from PSC diagnosis as early as 1972 onwards, and was upgraded to the EpiPSC2 prospective registry in 2016. The registry thoroughly applies case ascertainment and controls for duplicates. For a detailed description of case-finding and case-ascertainment, we refer to Boonstra et al. [1]. All eligible participants [alive and approachable in compliance with the General Data Protection Regulation (GDPR)] were invited to participate in the study by completing validated, digital patient-reported outcome (PRO) questionnaires sent every three months from May 2017 to May 2020. The study obtained approval from the institutional review board of the UMC Utrecht (reference: NL14614.041.06/METC 06-267/E).

### Data collection

At study inclusion, demographic and clinical characteristics were collected including age at diagnosis, sex, PSC type [i.e., large duct (LD), small duct (SD)], features of auto-immune hepatitis (AIH), concurrent diagnosis of IBD with corresponding date and type [i.e., Ulcerative Colitis (UC), Crohn’s disease (CD) and IBD-unspecified (IBD-u)], Alkaline Phosphatase (ALP) levels, LT with corresponding date and indication, occurrence of HB malignancies [i.e., cholangiocarcinoma (CCA), gallbladder carcinoma (GBC), and hepatocellular carcinoma (HCC)], deaths with corresponding date and cause, and hospital of inclusion. These data were supplemented annually from 2016 up to death, end of study (31st August 2020), or lost to follow-up date. CastorEDC, a cloud-based research data platform fully compliant with Good Clinical Practice standards, was used to collect and store data [13].

**Fig. 1:**
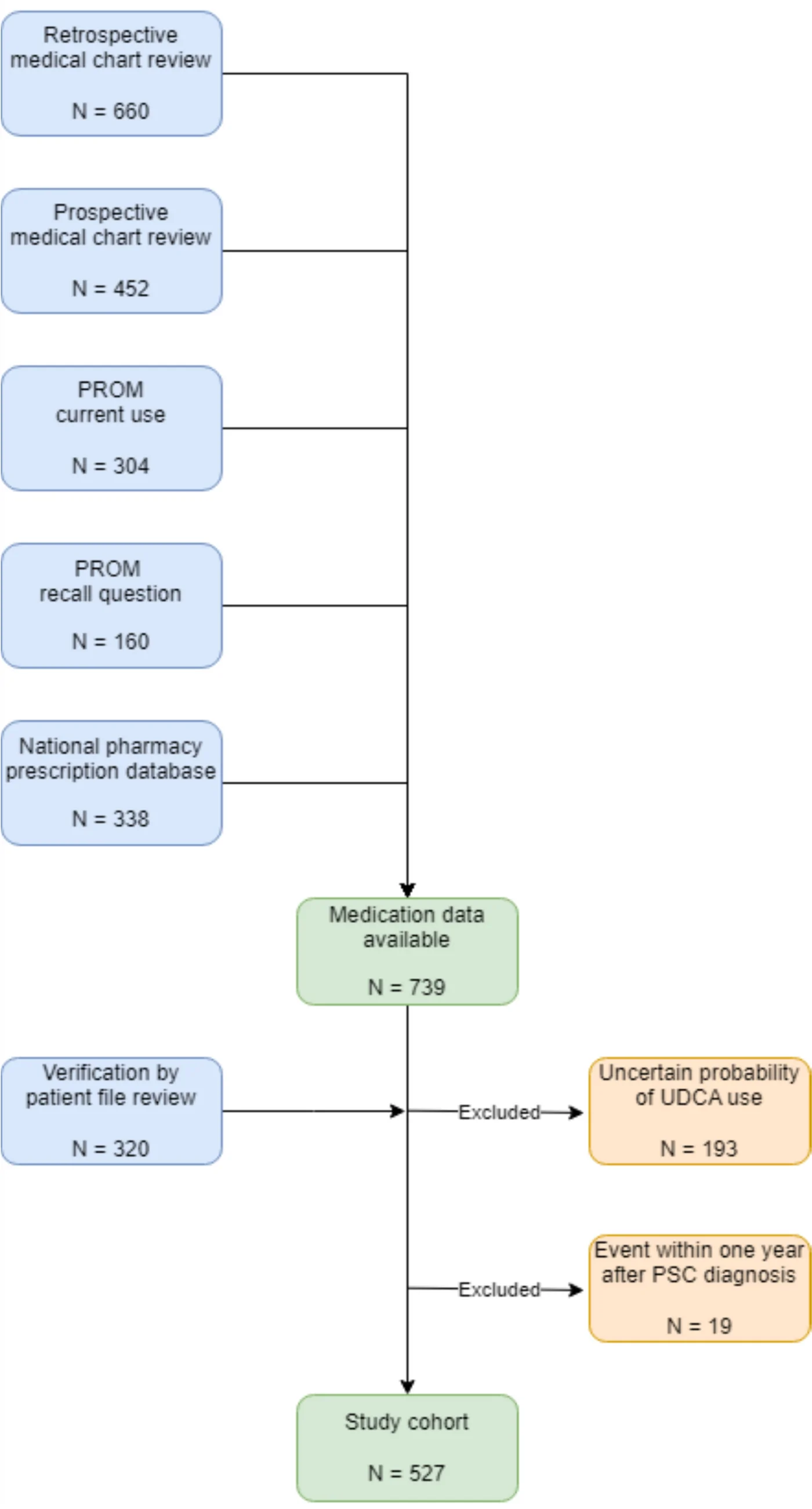
Flowchart of patient selection

Data regarding the use of UDCA were documented. Medication use was collected in five different manners, see Fig. 1; (1) retrospectively at inclusion on site by KB and CYP, (2) at last data collection within the EpiPSC/PBC study, (3) prospectively every three months using questionnaires, (4) recall questions within the prospective questionnaires, and (5) prescription data retrieved from SFK. Based on these five sources, use of UDCA was scored in terms of proportion of disease duration (i.e., never, less than 25%, between 25 and 50%, more than 50%, full duration, or unknown). Certainty (i.e., uncertain, reasonable, affirmative) of the medication use based on the different sources as well as patient charts was scored manually by CYP and BM, and if used for < 50% of disease duration whether this was primarily in the first or second half of disease duration. In case of a singular dispatch of medication, a patient was not categorized as a user. For those with missing medication data and consent for study participation, we manually checked medical records. Data regarding dosage could not be reliably obtained and were, therefore, excluded from the analyses. Only participants with reasonable or affirmative certainty of UDCA use were included in the analyses. Date of PSC diagnosis and start/stop-dates of UDCA use were used to determine proportion of use and if this was in the first or second half of the disease. A detailed description of the decision rules for scoring of medication use is listed as Supplementary File 1.

### Outcomes

Analyses were performed for the primary endpoints; LT or all-cause mortality (A) and LT, PSC-related death or occurrence of HB malignancy (B). PSC-related death was defined as death due to liver failure, HB malignancy, CRC, or other related cause. Sensitivity analyses were performed for the secondary endpoints; LT excluding those because of HB malignancy, or death due to liver failure (C); occurrence of HB malignancy (D).

### Statistical analysis

Continuous data with a normal distribution were expressed as means with standard deviation, skewed data were presented as median with interquartile range. Categorical variables were reported as absolute numbers with percentages. Categorical data were analyzed using the Pearson’s Chi-square test or the Fisher’s exact test in case of observed cell counts under ten. For continuous data, the choice between independent *t* test, one-way ANOVA, Mann–Whitney *U* test, or Kruskal–Wallis test depended on the number of groups and distribution of the data. Normality was evaluated through visual examination.

The effect of UDCA use was determined via the Cox proportional hazards model using the survival package in R [14]. Start of follow-up was defined as date of PSC diagnosis. All established risk factors, such as sex, age at diagnosis, large or small duct PSC, concurrent diagnosis of AIH, concurrent diagnosis of IBD, transplant center inclusion, and year of PSC diagnosis, were considered as covariates. UDCA use and IBD status were considered as time-dependent covariates by using the counting process approach [14]. This method divides the follow-up time of a participant in separate time intervals corresponding with the exposure status of a participant at that time. All participants with an event (i.e., diagnosis of HB malignancy, LT or death) within the first year after PSC diagnosis were excluded from the analyses, because it is assumed that events within 1 year from diagnosis could not have been modified by UDCA. Several sensitivity analyses were performed to evaluate the robustness of the results. First, we modeled a delay by assigning events occurring in the first year of initiating or discontinuing UDCA treatment to the preceding period. Second, we assessed the cumulative effect of UDCA by attributing cumulative years of UDCA use to the subsequent time periods. Third, we conducted an analysis comparing only those who had never used UDCA with those who had used it continuously throughout their disease. And lastly, sensitivity analyses were conducted to determine the impact of UDCA on either liver disease progression or occurrence of HB malignancies. Complete case analyses were performed. A *p* value less than 0.05 was considered statistically significant. Statistical analyses were conducted using IBM SPSS version 28.0 and R Studio [15, 16].

## Results

### Description of the study cohort

The study cohort comprised a total of 879 participants, with medication data available for 739 individuals. Patient characteristics of the entire study cohort and selection of individuals with available medication data were comparable (Supplementary Table 1). Data on retrospective medication use collected at study inclusion was available for 660 participants. Medication use at last data collection within the former EpiPSC/PBC study was available for 452 of the 653 EpiPSC/PBC participants. Out of the 511 individuals who were invited to participate in the prospective arm of the study, 359 (70%) consented to receive questionnaires. Among those, 316 (91%) completed at least one questionnaire. The questionnaires contained data on current medication use (304 participants), while 160 participants provided a recall date for start of UDCA use. Pseudonym and subsequent probabilistic data linkages with SFK resulted in 80,341 identified prescription records of 338 of 649 PSC-registry patients with 34,584 prescription records of relevant medications clusters (Supplementary Table 2) for 313 of those 338 patients. Dates of prescription ranged from 1998 to 2020. Verification of medication use by reviewing electronic patient files was performed for 320 participants. Due to an event within the first year after PSC diagnosis, 19 participants were excluded. The composition of the study population is depicted as a flowchart (Fig. 1).

### Patient characteristics by medication use

Probability of UDCA use was categorized as uncertain in 190 (27%), reasonably likely in 246 (34%), and affirmative in 281 (39%) participants. To maintain data accuracy, individuals with an uncertain probability of medication use were excluded from further analyses, resulting in a final study cohort of 527 patients. Participants categorized as uncertain use had an earlier calendar year of PSC diagnosis and were more frequently deceased compared to the remaining participants (Supplementary Tables 3, 4). The baseline characteristics of the three UDCA use groups are presented in Table 1, and resulted in 60 never users, 178 partial users, and 289 always users. Median calendar year of diagnosis was significantly earlier in the partial use group compared to never and always use groups (2000 vs 2004, vs 2006, respectively). Moreover, partial users were diagnosed at a younger age compared to never and always users (36 vs 41, vs 40, respectively). Alkaline phosphatase levels at diagnosis were available for almost half of the participants and did not differ between the groups. Clinical events and endpoints are presented in Table 2. CRC and HB malignancies were significantly more prevalent in the never use group compared to both user groups. Moreover, the median follow-up time in years was shortest in the never use group compared to the partial and always use groups (8 vs 14, vs 10, respectively).

**Table 1:** Baseline characteristics stratified by UDCA use

	Never (*N* = 60)	Partial (*N* = 178)	Always (*N* = 289)	*p* value
Male	37/60 (62%)	118/178 (66%)	191/289 (66%)	0.787
Age at diagnosis	41 (16)/54	36 (13)/178	40 (13)/286	0.006
Calendar year of PSC diagnosis	2004 (1995–2010)/59	2000 (1992–2006)/178	2006 (2000–2010)/289	< 0.001
Alkaline phosphatase at diagnosis	199 (122–305)/29	221 (152–339)/65	239 (159–367)/157	0.157
Transplant center inclusion	17/60 (28%)	40/178 (23%)	65/289 (23%)	0.600
Large duct	49/55 (82%)	164/178 (92%)	260/287 (90%)	0.748
Auto-immune hepatitis	2/53 (3%)	10/166 (6%)	26/265 (9%)	0.183
IBD	37/54 (62%)	134/178 (75%)	187/284 (65%)	0.100
Ulcerative colitis	28/37 (76%)	97/134 (72%)	137/187 (73%)	0.888
Crohn’s disease	8/37 (22%)	28/134 (21%)	41/187 (22%)	
IBD-unspecified	1/37 (3%)	9/134 (7%)	9/187 (5%)	
UDCA daily dose in milligrams	N.A	1000 (900–1350)/99	1000 (900–1250)/186	0.698

Data are *n*/*N* (%) or mean (SD)/*N* or median (IQR)/*N*

*N.A.* not applicable

**Table 2:** Clinical events and endpoints stratified by UDCA use

	Never (*N* = 60)	Partial (*N* = 178)	Always (*N* = 289)	*p* value
Follow-up in years (median [IQR])	8 (3–14)/54	14 (9–22)/178	10 (6–14)/286	< 0.001
Colorectal carcinoma	7/54 (12%)	6/178 (3%)	9/286 (3%)	0.004
Any HB malignancy	11/53 (18%)	20/177 (11%)	17/286 (6%)	0.002
Colectomy	9/60 (15%)	20/178 (11%)	37/289 (13%)	0.731
Liver transplantation	11/54 (18%)	45/178 (25%)	58/286 (20%)	0.429
Deceased	20/54 (33%)	44/178 (25%)	60/286 (21%)	0.038
PSC-related death	15/19 (75%)	30/39 (68%)	37/53 (62%)	0.639
Liver failure	2/15 (13%)	15/30 (50%)	14/37 (38%)	0.135
HB malignancy	9/15 (60%)	9/30 (30%)	16/37 (43%)	
Colorectal carcinoma	3/15 (20%)	2/30 (7%)	2/37 (5%)	

Data are *n*/*N* (%) or mean (SD)/*N* or median (IQR)/*N*

### Survival

During 6330 patient-years of follow-up, with a median of 11 (IQR 6–16) patient-years, 124 (24%) of the participants died. Of those, 82 (66%) died of a PSC-related cause with liver failure (34%) and HB malignancies (37%) as most common causes. A total of 114 LTs were observed with end-stage liver disease (61%) and recurrent cholangitis (33%) as most frequent indications. The median time up to LT or all-cause mortality was 21 (IQR 19–23) years. 48 HB malignancies were observed with a restricted mean time to occurrence of HB malignancy of 37 (IQR 35–39) years.

**Fig. 2:**
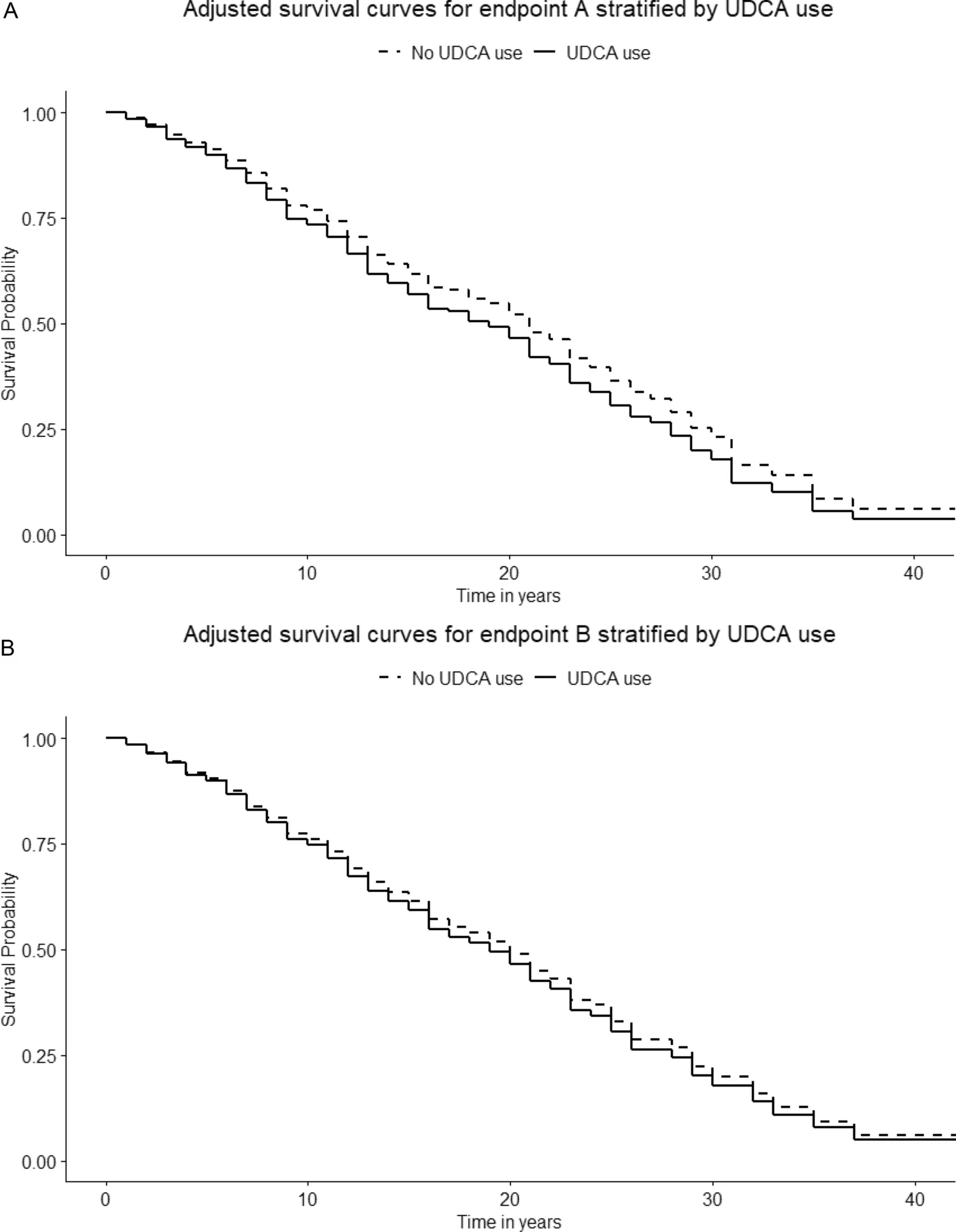
Adjusted survival curves for the endpoints LT or all-cause mortality (**A**), and LT, PSC-related death, or occurrence of HB malignancy (**B**) stratified by UDCA use

In the analysis evaluating survival regarding the endpoint LT or all-cause mortality, 501 participants with complete case information were included. The unadjusted hazard ratio (HR) of UDCA use compared to no use was 1.25 (95%CI 0.86–1.82). When corrected for known risk factors, the adjusted HR for UDCA use was 1.13 (95%CI 0.76–1.67). See also Fig. 2A. The results of both the univariable and multivariable models for the endpoint LT or all-cause mortality are presented in Table 3A. In the analyses focusing on the endpoint LT, PSC-related death or occurrence of HB malignancy, 502 participants were included. The unadjusted HR of UDCA use compared to no use was 1.19 (95%CI 0.81–1.76) and the adjusted HR was 1.07 (95%CI 0.71–1.60). See also Fig. 2B. Table 3B presents the results of both the univariable and multivariable models. For both endpoints, the HR for small duct PSC was significantly reduced, while the HRs for age and transplant center inclusion were significantly increased. Calendar year did not contribute significantly in both multivariable models.

**Table 3:** Multivariable risk for the endpoints LT or all-cause mortality (A) or LT, PSC-related death or occurrence of HB malignancy (B)

A. LT or all-cause mortality	Univariable HR (95%CI)	Multivariable HR (95%CI)
UDCA	1.25 (0.86–1.82)	1.13 (0.76–1.67)
Female	0.93 (0.69–1.25)	0.84 (0.62–1.15)
Age at diagnosis	1.03 (1.02–1.04)	1.03 (1.02–1.04)
Small duct PSC	0.27 (0.11–0.65)	0.30 (0.12–0.73)
Features of AIH	0.76 (0.40–1.44)	0.82 (0.42–1.58)
IBD status		
No IBD	Reference	Reference
Ulcerative colitis	1.30 (0.98–1.71)	1.21 (0.87–1.69)
Crohn’s disease	0.84 (0.55–1.29)	0.90 (0.56–1.45)
IBD-unspecified	0.61 (0.25–1.48)	0.63 (0.25–1.57)
Transplant center inclusion	1.64 (1.20–2.25)	1.67 (1.21–2.30)
Calendar year of PSC diagnosis	1.02 (1.00–1.04)	1.01 (0.99–1.03)

B. LT, PSC-related death, or occurrence of HB malignancy	Univariable HR (95%CI)	Multivariable HR (95%CI)
UDCA	1.19 (0.81–1.76)	1.07 (0.71–1.60)
Female	0.79 (0.57–1.10)	0.76 (0.54–1.05)
Age at diagnosis	1.02 (1.01–1.03)	1.02 (1.01–1.03)
Small duct PSC	0.24 (0.09–0.63)	0.25 (0.09–0.69)
Features of AIH	0.83 (0.44–1.58)	0.89 (0.46–1.71)
IBD status		
No IBD	Reference	Reference
Ulcerative colitis	1.28 (0.95–1.72)	1.18 (0.83–1.67)
Crohn’s disease	0.91 (0.59–1.39)	0.93 (0.57–1.53)
IBD-unspecified	0.53 (0.19–1.42)	0.56 (0.20–1.56)
Transplant center inclusion	1.61 (1.16–2.25)	1.57 (1.12–2.20)
Calendar year of PSC diagnosis	1.01 (0.99–1.03)	1.01 (0.99–1.03)

### Sensitivity analyses

First, we modeled a delayed effect of UDCA use for the endpoint LT, PSC-related death, or occurrence of HB malignancy. When incorporating a delay of 1 year, the adjusted HR of UDCA use was 1.04 (95%CI 0.60–1.81). The results of the multivariable model are presented as Supplementary Table 5A.

Second, we assessed the cumulative effect of UDCA for the endpoint LT, PSC-related death, or occurrence of HB malignancy. When incorporating a cumulative measure of UDCA use in years, the adjusted HR of UDCA use was 1.16 (95%CI 0.77–1.74) and the HR for the cumulative years of UDCA use was 0.99 (95%CI 0.98–1.00) with a *p* value of 0.02. The results of the multivariable model are presented as Supplementary Table 5B.

Third, we compared the never UDCA users with those who had used it continuously throughout their disease for the endpoint LT, PSC-related death, or occurrence of HB malignancy. 329 participants were included in this analysis. The adjusted HR for continuous UDCA use compared to never use was 0.79 (95%CI 0.47–1.33; *p* value = 0.37). The results of the multivariable model are presented as Table 4.

**Table 4:** Multivariable risk for the endpoint LT, PSC-related death, or occurrence of HB malignancy incorporating a cumulative effect of UDCA use limited to only never users (*n* = 60) and those (*n* = 279) who had used it throughout their complete disease course

	Multivariable HR (95%CI)
UDCA	0.79 (0.47–1.33)
Cumulative years of UDCA use	0.99 (0.98–1.00)
Female	0.75 (0.48–1.16)
Age at diagnosis	1.02 (1.01–1.04)
Small duct PSC	0.30 (0.09–0.95)
Features of AIH	0.88 (0.40–1.93)
IBD status	
No IBD	Reference
Ulcerative colitis	1.46 (0.93–2.30)
Crohn’s disease	0.93 (0.47–1.85)
IBD-unspecified	0.32 (0.04–2.39)
Transplant center inclusion	1.53 (1.00–2.35)
Calendar year of PSC diagnosis	0.99 (0.97–1.02)

And finally, sensitivity analyses were conducted to determine the impact of UDCA use on either liver disease progression or occurrence of HB malignancies. For the endpoint LT (excluding those because of HB malignancy) or death due to liver failure, 502 participants were included in the analysis. The adjusted HR for UDCA use was 1.51 (95%CI 0.89–2.58) compared to no use. Supplementary Table 6A presents the results of the multivariable model. For the endpoint occurrence of HB malignancy, 505 participants were included in the analysis. The adjusted HR for UDCA use was 0.68 (95%CI 0.34–1.37). The results of the multivariable model are presented in Supplementary Table 6B.

## Discussion

Despite decades of prescription and research, the long-term beneficial effects of UDCA have never been shown, apart from one retrospective series in Japan [17].

The vast majority of literature data do not support the use of UDCA to improve transplant-free survival. In 2009, a high-dose UDCA trial was prematurely stopped, because the risk of the primary endpoint was 2.3 times higher in the 28–30 mg/kg day UDCA-treated group [18]. A Scandinavian trial also failed to show benefit of 5-year treatment with 17–23 mg/kg UDCA versus placebo on death or liver transplantation [19]. However, this was markedly underpowered to detect a positive contrast on solid clinical endpoints [20]. A post hoc analysis extending the follow-up of the Scandinavian trial encompassing 10 years of follow-up, too failed to show a protective effect of UDCA on transplant-free survival [8].

A meta-analysis on 8 RCTs comprising 567 patients treated for 3 months to 6 years with doses ranging from 10 to 30 mg/kg/day found no significant difference in mortality (OR = 0.6, 95%CI 0.4–1.4), nor in mortality or liver transplantation (OR = 0.84, 95%CI 0.5–1.4) [21]. Although a trend was seen in favor of the UDCA-treated group, incidence of CCA did not differ between groups. The total of 2300 patient-years in these RCTs was rather low to detect an effect on solid clinical endpoints [20]. Hence, since obtaining high-quality RCT data for demonstrating or refuting the beneficial effect of UDCA is not feasible, the field has to rely on observational data. However, long-term population-based data are limited. In a nationwide retrospective study from Sweden, time-varying Cox regression yielded an increased risk for death or liver transplantation and hepatobiliary cancer for those ever prescribed UDCA [22].

We present the largest observational study to date showing no apparent effect of UDCA treatment on PSC-related death, liver transplantation, or occurrence of HB malignancy. Our data show a less-pronounced association on long-term survival in terms of PSC-related mortality, liver transplant, and hepatobiliary cancer compared to the Swedish data. Conversely, our findings for the endpoint liver transplantation or death due to liver failure align with those reported by Stokkeland et al., who observed that UDCA use may be associated with an elevated risk of adverse events.

Reasons why UDCA despite its anticholestatic effects is not associated with long-term benefit in PSC remain speculative. Cholestasis impairs absorption of UDCA, which may result in metabolization to hepatotoxic bile acids such as lithocholic acid (LCA) by the gut microbiota [23–25]. While, in a mouse model, LCA had anti-inflammatory properties in the colon, clear cytotoxic effects from LCA were seen in bile ductules in a rat model [24, 25]. It is conceivable that potentially beneficial effects of UDCA shift to adverse off-target effects in advanced cholestasis.

The discrepancy of our results with the seminal paper of Arizumi et al. from Japan is striking [17]. There may be several explanations for this difference: Compared to the size of the Japanese population, the study contained a relatively small number of patients, which runs the risk that the cohort may not have been sufficiently representative of the PSC population at large. The mean follow-up time was 5.1 years versus 11.8 years in our study. Patients with small duct PSC were excluded in the Japanese study, which may have skewed their cohort to more severe patients. Immortality bias which is in our view a potentially important confounder was not taken into account in the Japanese study. In addition, duration of UDCA use was not considered. Notwithstanding, it cannot be ruled out that PSC in Japan behaves differently than in Western-Europe.

Although underpowered, our sensitivity analysis shows a non-significant association between UDCA use and reduced incidence of HB malignancy. In pre-clinical models, UDCA has shown chemopreventive properties on HCC, CCA, and CRC cell lines [26–30]. In humans, several retrospective studies indicated that the risk of CRC is reduced in PSC-UC patients using UDCA [31, 32]. However, long-term high-dose UDCA use was reported to be associated with an increased risk of colonic neoplasia [33]. A meta-analysis showed no significant reduced risk [relative risk 0.87 (95%CI 0.51–1.49)] on the development of CRC in patients with PSC and IBD [34]. Observational data on the impact of UDCA treatment on the occurrence of CCA are scarce [35].

### Strengths and limitations

Observational research is inherently subject to bias, such as heterogeneity of included cases, referral bias, missing data, overlooked confounders, etc. Here, all cases have undergone case-ascertainment at the source by three experienced investigators (CYP, KB and KvM). We have corrected for transplantation-center bias in our analysis. Furthermore, we have tried to take as many potential confounders into account including well-known such as sex, PSC type, age at diagnosis, concurrent IBD and type of, but—importantly—also immortality bias by incorporating UDCA as time-dependent variable. A putative different effect of UDCA in patients who used the drug earlier in their disease course versus later was made less likely by a sensitivity analysis comparing partial users who predominantly were prescribed UDCA during the first half of their disease course versus second half. This did not reveal a difference in effect size (data not shown). Because we could mine four different sources of medication use and applied two different decision rules for estimating the duration of medication use, and discarded in the end patients with uncertain use, we think that user classification was as optimal as attainable. To counter selection bias, calendar year of PSC diagnosis was considered as covariate. As UDCA became common practice as prescription for PSC patients in the late nineties, patients who had an earlier calendar year of PSC diagnosis could potentially represent a selection of patients with a more favorable prognosis as they have lived long enough to participate in the study and receive the treatment when it became available. This potential bias was not apparent as calendar year did not significantly contribute to the multivariable models. Since ALP levels at diagnosis were comparable for all groups, there was no indication that prescription bias played a substantial role. In the Netherlands, commonly, every patient with a diagnosis of PSC gets UDCA prescribed irrespective of the severity of cholestasis. Nevertheless, prescription bias cannot fully be ruled out, and would perhaps offer an alternative explanation for the observed trend benefiting the always-user group over the never-user group, suggesting that physicians were possibly less inclined to prescribe UDCA to patients with a less favorable prognosis. For instance, the occurrence of HB carcinoma in never users was 18% versus 6% in always users. It may be that physicians refrained from prescribing a drug like UDCA aimed at impacting long-term prognosis in these patients. Excluded patients categorized as uncertain use, which we deliberately left out of the analysis to avoid the inherent bias of retrospective data, were more frequently included in district general hospitals, which could lead to underrepresentation of these patients. Data on dosing were reliably available for all prospectively followed patients. However, because a putative modifying effect on outcome was presumed upon long-term use only, we chose to involve all patients from whom we had reasonable or affirmative medication use available with the longest follow-up attainable. Indeed, our study presents the longest FU data regarding UDCA use so far. A drawback was that reliable granular information on dosing was available in half of the cohort only. However, UDCA dosing in the Netherlands since its introduction has been commonly 10–20 mg/kg, which is according to most guidelines on PSC, despite the fact that there is no evidence on the optimal dose of UDCA in PSC.

In conclusion, this population-based long-term observational study failed to demonstrate that use of UDCA affects long-term outcomes in PSC patients, and consequently does not support current suggestions in recent clinical practice guidelines.

## Supplementary Information

Below is the link to the electronic supplementary material.

Supplementary file1 (DOCX 45 KB)
