# Supplementary material for: The longstanding issue of the benefit of ursodeoxycholic acid in primary sclerosing cholangitis: a long-term population-based study
Source: J Gastroenterol. 2026 Jun 15;61(11):1643–52. doi: 10.1007/s00535-026-02449-7 (PMC13695685; doi:10.1007/s00535-026-02449-7)
Supplement: Supplementary file 1 [file 535_2026_2449_MOESM1_ESM.docx]

**Supplementary file 1**

**Decision rules for medication use**

Medication use was scored based on available data from multiple sources: retrospective data from the retrospective EpiPSCPBC registry, retrospective and prospective data from the EpiPSC2 registry, data from prospective questionnaires containing recall data and current use, as well as data from *Stichting Farmaceutische Kerngetallen* which were pseudonym linked. The three types of medication which were manually scored were ursodeoxycholic acid, thiopurines and NSAID’s.

The first step in the manual scoring was to allocate a score for the proportion of use (relative to the disease duration), the probability or certainty of this score and if the patient had used the respective drug in the first half of his/her disease course. Scoring was performed by BM and CP. Based on the available data the following decision rules were applied:

Proportion of use:
0 = never
1 = <25% of PSC duration
2 = 25%<x<50% of PSC duration
3 = >50% of PSC duration
4 = full PSC duration
99 = unknown

Disease duration was determined by the date of PSC diagnosis and either date of first liver transplantation, death or date of data extraction (August 2020). In case of missing data regarding medication use at the start or end of the disease duration, the minimal observed duration was scored. In case of missing data in between periods of known medication use, the proportion was scored according to the data available at the start and end of follow-up, assuming that UDCA use was continuous and that patients did not use the medication intermittently:

No use at start and no use at the end = never or unknown
Use at the start and use at the end = full PSC duration or unknown
No use at start and use at the end = proportion known use at end of follow-up or unknown
Use at start and no use at the end = proportion known use at start of follow-up or unknown

The amount of missing data was taken into consideration when defining one of aforementioned proportions or scoring the proportion as unknown.

Probability or certainty of the data:
1 = uncertain
2 = reasonable
3 = affirmative

Uncertain was scored if all or the majority of the data was missing. Reasonable was scored if the data was incomplete, although a sufficient amount of data from at least two sources was available to assess use or non-use. Affirmative was scored if all data was available or if the available data covered the full disease duration.

Use in the first half of the disease duration:
0 = no
1 = yes
99 = unknown

Disease duration was determined for three time periods; 1) from the date of PSC diagnosis up to either date of death or date of data extraction (August 2020), 2) from date of PSC diagnosis up to either date of death, liver transplantation, or date of data extraction, and 3) from date of PSC diagnosis up to the date of diagnosis of a hepatobiliary malignancy, death or date of data extraction. Based on the disease duration and the date of diagnosis, the approximate first half of disease duration was deduced. As a general rule, if the proportion of use was >50% a patient had used medication in the first half of the disease duration.

In the second step of the scoring process, patients with a substantial part of data missing or inconsistencies in their data were checked manually in their respective medical files (if accessible). The proportion, probability and use in the first half of the disease duration were adjusted accordingly. Scoring was performed by BM.

**Determining start and stop dates**

Start and stop dates were determined based on the proportion of use and whether medication use was in the first or second half of their disease duration.

In the "never use" and "full disease duration use" groups, the entire follow-up period was coded as non-use and use, respectively. For participants who used UDCA for more than half of their disease duration, 75% of the disease duration was coded as use, and the remaining 25% as non-use. Since discontinuation of UDCA after prolonged use is uncommon, we designated the first 25% of the disease duration as non-use and the last 75% as use. For participants who used UDCA for 25% to 50% of their disease duration, 50% of the disease duration was coded as use and 50% as non-use, depending on whether the use occurred in the first or second half of the disease course. For participants who used UDCA for less than 25% of their disease duration, 25% was coded as use and 75% as non-use. If the medication was used in the first half of the disease, the first 25% was coded as use; if the use occurred in the second half, the last 25% was coded as use.

**Supplementary table 1. Patient characteristics of the complete cohort and selection of patients with available medication data**

|  | Complete cohort (N=879) | Medication data availlable (N=739) | |
| --- | --- | --- | --- |
| Male | 558 (64%) | 470 (64%) | |
| Age at diagnosis (mean [sd]) | 39 (15) | 39 (15) | |
| Follow- up in years (median [IQR]) | 13 (8-20) | 13 (8-20) | |
| Calendar year of PSC diagnosis (median [IQR]) | 2003 (1995-2007) | 2003 (1995-2008) | |
| Alkaline phosphatase at diagnosis (median [IQR]) | 239 (163-407) | 240 (161-395) | |
|  |  |  | |
| Liver transplantation | 204 (23%) | 179 (25%) | |
| Deceased | 238 (27%) | 235 (32%) | |
| PSC-related death | 152 (64%) | 151 (64%) |  |
| Liver failure | 54 (36%) | 54 (36%) |  |
| HB malignancy | 66 (43%) | 65 (43%) |  |
| Transplant centre inclusion | 191 (22%) | 182 (25%) | |
| Periferal centre inclusion | 277 (32%) | 207 (28%) | |
|  |  |  | |
| Large duct PSC | 773 (88%) | 661 (89%) | |
| Auto-immune hepatitis | 44 (5%) | 43 (6%) | |
| IBD | 628 (71%) | 526 (71%) | |
| Ulcerative colitis | 461 (73%) | 385 (73%) | |
| Crohn’s disease | 124 (20%) | 105 (20%) | |
| IBD-u | 32 (5%) | 25 (5%) | |
| Colectomy | 103 (12%) | 92 (12%) | |
|  |  |  | |
| Colorectal carcinoma | 36 (4%) | 33 (5%) | |
| Any HB malignancy | 88 (10%) | 84 (12%) | |

**Supplementary table 2: Relevant medication groups**

ursodeoxycholic acid

mesalazine

thiopurines

methotrexate

NSAIDs

biologicals

corticosteroiden

anti-pruritica

statins

protonpump inhibitors

treatment for liver cirrhosis

rifaximin

furosemide

spironolacton

lactulose

norfloxacin

immunesuppressants

ciclosporin

tacrolimus

mycofenolate mofetil

non-selective beta blockers

loperamide

**Supplementary table 3. Patient characteristics of the participants with reasonable or affirmative UDCA use and participants with uncertain UDCA use**

|  | Participants with reasonable or affirmative UDCA use (N=527) | Participants with uncertain UDCA use (N=190) | | P-value | |
| --- | --- | --- | --- | --- | --- |
| Male | 346 (66) | 112 (59) | | 0.099 | |
| Age at diagnosis (mean [sd]) | 39 (14) | 40 (17) | | 0.531 | |
| Follow- up in years (median [IQR]) | 11 (7-16) | 9 (6-16) | | 0.245 | |
| Calendar year of PSC diagnosis (median [IQR]) | 2004 (1997-2008) | 1998 (1992-2004) | | <.001 | |
| Alkaline phosphatase at diagnosis (median [IQR]) | 224 (152-357) | 261 (197-566) | | 0.125 | |
|  |  |  | |  | |
| Liver transplantation | 114 (22) | 57 (30) | | 0.017 | |
| Deceased | 124 (24%) | 90 (47%) | | <.001 | |
| PSC-related death | 82 | 48 | 0.237 | |  |
| Liver failure | 31 | 20 | 0.633 | |  |
| HB malignancy | 34 | 16 | 0.633 | |  |
| Transplant centre inclusion | 122 (23) | 53 (28) | | 0.192 | |
|  |  |  | |  | |
| Large duct PSC | 473 (90) | 166 (87) | | 0.342 | |
| Auto-immune hepatitis | 38 (7) | 4 (2) | | 0.076 | |
| IBD | 379 (72) | 135 (71) | | 1.000 | |
| Ulcerative colitis | 269 | 106 | | 0.280 | |
| Crohn’s disease | 79 | 24 | | 0.280 | |
| IBD-u | 21 | 4 | | 0.280 | |
| Colectomy | 74 (14) | 31 (16) | | 0.447 | |
|  |  |  | |  | |
| Colorectal carcinoma | 22 (4) | 9 (5) | | 0.725 | |
| HB malignancy | 48 (9) | 18 (10) | | 0.832 | |

**Supplementary table 4. Patient characteristics stratified by UDCA use**

|  | Never (N=60) | <25% (N=14) | 25%<50% (N=53) | >50% (N=111) | Full disease (N=289) | P-value | |
| --- | --- | --- | --- | --- | --- | --- | --- |
| Male | 37 (62%) | 6 (43%) | 32 (60%) | 80 (72%) | 191 (66%) | 0.174 | |
| Age at diagnosis (mean [sd]) | 41 (16) | 38 (15) | 38 (14) | 36 (12) | 40 (13) | 0.026 |  |
| Follow- up in years (median [IQR]) | 8 (3-14) | 12 (4-19) | 14 (8-24) | 14 (10-23) | 10 (6-14) | <.001 | |
| Calendar year of PSC diagnosis (median [IQR]) | 2004 (1995-2010) | 2004 (2001-2012) | 1998 (1988-2006) | 1999 (1992-2006) | 2006 (2000-2010) | <.001 | |
| Alkaline phosphatase at diagnosis (median [IQR]) | 199 (122-305) | 584 (163-715) | 278 (200-323) | 176 (129-297) | 239 (159-367) | 0.030 | |
| Transplant centre inclusion | 17 (28%) | 2 (14%) | 15 (28%) | 23 (21%) | 65 (23%) | 0.600 | |
|  |  |  |  |  |  |  | |
| Large duct PSC | 49 (82%) | 12 (86%) | 49 (93%) | 103 (93%) | 260 (90%) | 0.853 | |
| Auto-immune hepatitis | 2 (3%) | 0 (0%) | 1 (2%) | 9 (8%) | 26 (9%) | 0.198 | |
| IBD | 43 (72%) | 8 (57%) | 43 (81%) | 85 (77%) | 200 (69%) | 0.226 | |
| Ulcerative colitis | 28 (65%) | 6 (75%) | 30 (70%) | 61 (72%) | 144 (72%) | 0.766 | |
| Crohn’s disease | 8 (19%) | 1 (13%) | 8 (19%) | 20 (24%) | 42 (21%) | 0.766 | |
| IBD-u | 1 (2%) | 1 (12%) | 5 (12%) | 4 (5%) | 10 (5%) | 0.766 | |
| Colorectal carcinoma | 7 (12%) | 0 (0%) | 2 (4%) | 4 (4%) | 9 (3%) | 0.020 | |
| Any HB malignancy | 11 (18%) | 2 (14%) | 3 (6%) | 15 (14%) | 17 (6%) | 0.004 | |
| Colectomy | 10 (17%) | 2 (14%) | 9 (17%) | 13 (12%) | 40 (14%) | 0.873 | |
|  |  |  |  |  |  |  | |
| Liver transplantation | 11 (18%) | 2 (14%) | 12 (23%) | 31 (28%) | 58 (20%) | 0.501 | |
| Deceased | 20 (33%) | 2 (14%) | 12 (23%) | 30 (27%) | 60 (21%) | 0.099 | |
| PSC-related death | 15 (75%) | 1 (50%) | 7 (58%) | 22 (%) | 37 (%) | 0.486 | |
| Liver failure | 2 (%) | 0 (%) | 5 (%) | 10 (%) | 14 (%) | 0.341 | |
| HB malignancy | 9 (%) | 1 (%) | 1 (%) | 7 (%) | 16 (%) | 0.341 | |
| Colorectal carcinoma | 3 (%) | 0 (%) | 1 (%) | 1 (%) | 2 (%) | 0.341 | |

| **Supplementary Table 5. Multivariable risk for the endpoint LT, PSC-related death or occurrence of HB malignancy incorporating delay (A) and a cumulative effect of UDCA use (B)** | | |
| --- | --- | --- |
| **A. LT, PSC-related death or occurrence of HB malignancy** |  | Multivariable  HR (95%CI) |
| UDCA with a delayed effect of one year |  | 1.04 (0.60-1.81) |
| Female |  | 0.91 (0.58-1.42) |
| Age at diagnosis |  | 1.04 (1.02-1.06) |
| Small duct PSC |  | 0.28 (0.07-1.16) |
| Features of AIH |  | 0.43 (0.10-1.79) |
| IBD status | No IBD  Ulcerative colitis Crohn’s disease IBD-unspecified | Reference  1.33 (0.81-2.21)  1.16 (0.58-2.30)  1.02 (0.35-2.99) |
| Transplant centre inclusion |  | 1.23 (0.73-2.06) |
| Calendar year of PSC diagnosis |  | 1.00 (0.97-1.04) |
| **B. LT, PSC-related death or occurrence of HB malignancy** |  | Multivariable  HR (95%CI) |
| UDCA |  | 1.16 (0.77-1.74) |
| Cumulative years of UDCA use |  | 0.99 (0.98-1.00) |
| Female |  | 0.77 (0.55-1.07) |
| Age at diagnosis |  | 1.02 (1.01-1.03) |
| Small duct PSC |  | 0.26 (0.10-0.72) |
| Features of AIH |  | 0.86 (0.44-1.66) |
| IBD status | No IBD  Ulcerative colitis  Crohn’s disease  IBD-unspecified | Reference  1.10 (0.77-1.55)  0.86 (0.52-1.41)  0.50 (0.18-1.39) |
| Transplant centre inclusion |  | 1.55 (1.11-2.17) |
| Calendar year of PSC diagnosis |  | 1.01 (0.99-1.03) |

| **Supplementary Table 6. Multivariable risk for the endpoints LT excluding those because of HB malignancy, or death due to liver failure (A); occurrence of HB malignancy (B)** | | |
| --- | --- | --- |
| **A. LT (excluding those because of HB malignancy) or death due to liver failure** |  | Multivariable  HR (95%CI) |
| UDCA |  | 1.51 (0.89-2.58) |
| Female |  | 0.69 (0.46-1.03) |
| Age at diagnosis |  | 1.02 (1.00-1.04) |
| Small duct PSC |  | 0.19 (0.05-0.79) |
| Features of AIH |  | 1.11 (0.54-2.25) |
| IBD status | No IBD  Ulcerative colitis Crohn’s disease IBD-unspecified | Reference  1.06 (0.71-1.60)  0.70 (0.37-1.32)  0.41 (0.10-1.71) |
| Transplant centre inclusion |  | 2.15 (1.46-3.15) |
| Calendar year of PSC diagnosis |  | 1.00 (0.98-1.03) |
| **B. Occurrence of HB malignancy** |  | Multivariable  HR (95%CI) |
| UDCA |  | 0.68 (0.34-1.37) |
| Female |  | 0.95 (0.51-1.77) |
| Age at diagnosis |  | 1.02 (0.99-1.04) |
| Small duct PSC |  | 0.54 (0.13-2.30) |
| Features of AIH |  | 0.00 (0.00-N.A.)^a^ |
| IBD status | No IBD  Ulcerative colitis  Crohn’s disease  IBD-unspecified | Reference  1.59 (0.79-3.19)  1.53 (0.62-3.78)  1.35 (0.30-6.13) |
| Transplant centre inclusion |  | 0.55 (0.24-1.24) |
| Calendar year of PSC diagnosis |  | 1.00 (0.96-1.05) |
| ^a^Confidence interval could not be computed due to zero cell count | | |

**Supplementary STROBE checklist**

STROBE Statement—Checklist of items that should be included in reports of ***cohort studies***

|  | | Item No | Recommendation | Page No |
| --- | --- | --- | --- | --- |
| **Title and abstract** | | 1 | (*a*) Indicate the study’s design with a commonly used term in the title or the abstract | 1,3 |
|  |  |  | (*b*) Provide in the abstract an informative and balanced summary of what was done and what was found | 3 |
| Introduction | | | | |
| Background/rationale | | 2 | Explain the scientific background and rationale for the investigation being reported | 4 |
| Objectives | | 3 | State specific objectives, including any prespecified hypotheses | 4,5 |
| Methods | | | | |
| Study design | | 4 | Present key elements of study design early in the paper | 5 |
| Setting | | 5 | Describe the setting, locations, and relevant dates, including periods of recruitment, exposure, follow-up, and data collection | 5 |
| Participants | | 6 | (*a*) Give the eligibility criteria, and the sources and methods of selection of participants. Describe methods of follow-up | 5 |
|  |  |  | (*b*) For matched studies, give matching criteria and number of exposed and unexposed | N.A. |
| Variables | | 7 | Clearly define all outcomes, exposures, predictors, potential confounders, and effect modifiers. Give diagnostic criteria, if applicable | 5,6,7 |
| Data sources/ measurement | | 8* | For each variable of interest, give sources of data and details of methods of assessment (measurement). Describe comparability of assessment methods if there is more than one group | 5,6,7 |
| Bias | | 9 | Describe any efforts to address potential sources of bias | 5,6,7 |
| Study size | | 10 | Explain how the study size was arrived at | 5 |
| Quantitative variables | | 11 | Explain how quantitative variables were handled in the analyses. If applicable, describe which groupings were chosen and why | 6 |
| Statistical methods | | 12 | (*a*) Describe all statistical methods, including those used to control for confounding | 6,7 |
|  |  |  | (*b*) Describe any methods used to examine subgroups and interactions | 6.7 |
|  |  |  | (*c*) Explain how missing data were addressed | 6.7 |
|  |  |  | (*d*) If applicable, explain how loss to follow-up was addressed | 5 |
|  |  |  | (*e*) Describe any sensitivity analyses | 6.7 |
| Results | | | |  |
| Participants | | 13* | (a) Report numbers of individuals at each stage of study—eg numbers potentially eligible, examined for eligibility, confirmed eligible, included in the study, completing follow-up, and analysed | 7,8 |
|  |  |  | (b) Give reasons for non-participation at each stage | 7,8 |
|  |  |  | (c) Consider use of a flow diagram | Figure 1 |
| Descriptive data | | 14* | (a) Give characteristics of study participants (eg demographic, clinical, social) and information on exposures and potential confounders | 7,8 Table 1 |
|  |  |  | (b) Indicate number of participants with missing data for each variable of interest | 7,8 Table 1 |
|  |  |  | (c) Summarise follow-up time (eg, average and total amount) | 8 |
| Outcome data | | 15* | Report numbers of outcome events or summary measures over time | 8 |
| Main results | 16 | (*a*) Give unadjusted estimates and, if applicable, confounder-adjusted estimates and their precision (eg, 95% confidence interval). Make clear which confounders were adjusted for and why they were included | | 8.9 |
|  |  | (*b*) Report category boundaries when continuous variables were categorized | | N.A. |
|  |  | (*c*) If relevant, consider translating estimates of relative risk into absolute risk for a meaningful time period | | N.A. |
| Other analyses | 17 | Report other analyses done—eg analyses of subgroups and interactions, and sensitivity analyses | | 8,9 |
| Discussion | | | | |
| Key results | 18 | Summarise key results with reference to study objectives | | 9 |
| Limitations | 19 | Discuss limitations of the study, taking into account sources of potential bias or imprecision. Discuss both direction and magnitude of any potential bias | | 10,11 |
| Interpretation | 20 | Give a cautious overall interpretation of results considering objectives, limitations, multiplicity of analyses, results from similar studies, and other relevant evidence | | 9-11 |
| Generalisability | 21 | Discuss the generalisability (external validity) of the study results | | 9-11 |
| Other information | | | | |
| Funding | 22 | Give the source of funding and the role of the funders for the present study and, if applicable, for the original study on which the present article is based | | 2 |

*Give information separately for exposed and unexposed groups.

**Note:** An Explanation and Elaboration article discusses each checklist item and gives methodological background and published examples of transparent reporting. The STROBE checklist is best used in conjunction with this article (freely available on the Web sites of PLoS Medicine at http://www.plosmedicine.org/, Annals of Internal Medicine at http://www.annals.org/, and Epidemiology at http://www.epidem.com/). Information on the STROBE Initiative is available at http://www.strobe-statement.org.
